# Thermodynamic and Kinetic Aspects of Formamidinium Lead Iodide Thermal Decomposition

**DOI:** 10.1021/acs.jpcc.1c06729

**Published:** 2021-09-30

**Authors:** Alessio Luongo, Bruno Brunetti, Stefano Vecchio Ciprioti, Andrea Ciccioli, Alessandro Latini

**Affiliations:** † Dipartimento di Chimica, Sapienza Università di Roma, Piazzale Aldo Moro 5, Roma 00185, Italy; ‡ Consiglio Nazionale delle Ricerche - Istituto per lo Studio dei Materiali Nanostrutturati, c/o Dipartimento di Chimica, Sapienza Università di Roma, Piazzale Aldo Moro 5, 00185 Roma, Italy; § Dipartimento S.B.A.I., Sapienza Università di Roma, Via del Castro Laurenziano 7, Roma 00161, Italy

## Abstract

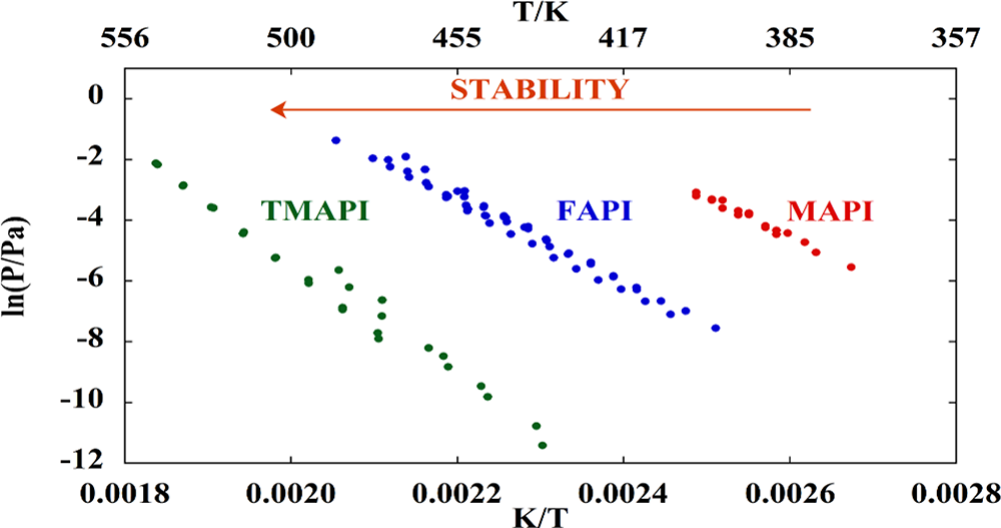

We report the results of a multi-technique study on the thermodynamics and kinetics of formamidinium lead iodide (FAPI) thermal decomposition. Thermodynamics was investigated by means of Knudsen effusion techniques. Kinetics was studied either by temperature-controlled powder X-ray diffraction or by two isoconversional treatments of differential scanning calorimetry data. FAPI appears to be much more thermally stable compared to methylammonium lead iodide, as predictable from the lower acidity of the formamidinium cation compared to methylammonium. The chemical processes responsible for its thermal degradation appear to be quite complex as highlighted by the composition of the gaseous phase evolved during the process. The apparent activation energy values of the decomposition obtained from X-ray diffraction (XRD) (112 ± 9 kJ/mol) and differential scanning calorimetry (DSC) measurements (205 ± 20 and 410 ± 20 kJ/mol, respectively, for the first and second decomposition steps identified by the deconvolution procedure) reflect the different steps of the process observed by the two techniques. The thermodynamic properties of the more important decomposition channels and the enthalpy of formation of FAPI were estimated by combining the results of Knudsen effusion measurements.

## Introduction

Perovskite solar cells have been representing the fastest growing photovoltaic technology in terms of conversion efficiency improvement since their first appearance in 2009.^1,2^ Methylammonium lead iodide (MAPI) emerged as the first and prototypical compound of the hybrid perovskite family. Although known for decades,^3−5^ its exceptional and rather unique semiconducting properties (gap value and type, defect tolerance) drew the attention of the research community only in the last decade.^6−8^ As soon as its very high performances in photovoltaic devices were noticed, its limited chemical and thermal stability rose as a very serious problem. More generally, the relatively poor thermal and chemical stability of hybrid halide perovskites for photovoltaic applications in real operating conditions is still the main obstacle to their commercial implementation. MAPI has been extensively studied in all its weak points with both experimental and theoretical approaches, and consequently, some stabilization procedures, though rather unsuccessful, have been developed to circumvent its stability problems. Among compounds with similar properties, cesium lead iodide and formamidinium lead iodide, FAPI is regarded as the most important and extensively studied one,^9^ pure or in solid solutions with MAPI, for the development of more performant and more stable devices.^10−13^

Black (i.e., photoactive) phases of cesium lead iodide and FAPI are stable only at relatively high temperatures (*T* > 320 °C for CsPbI_3_^14^ and *T* > 185 °C for FAPI^15^). Conversely, at room temperature, they are yellow solids not suitable for photovoltaic use. Nevertheless, strategies to stabilize the black phases at room temperature have been developed to use them in photovoltaic cells.^14,16^

Coming now to consider the stability issues, while a number of papers have been devoted to the intrinsic stability of MAPI^17−22^ and CsPbI_3_,^23−26^ few data are currently available on FAPI. In particular, data about the thermodynamics of its decomposition, as well as a detailed kinetic analysis of the process, are totally lacking in the literature. Such data are of crucial importance for technological applications, such as photovoltaics, to predict the stability of the material in real operating conditions, as well as its compatibility with the other materials comprising the devices. Besides, the knowledge of these data is exploitable in aiming at developing material stabilization and/or protection strategies. Our efforts in this work were focused to fill part of this gap. To this end, we applied a multi-technique strategy aimed at investigating both the kinetic and the thermodynamic aspects of the decomposition processes of FAPI at moderate temperatures.

The structural evolution and the activation energy associated with the nucleation and growth of the solid decomposition product (PbI_2_) have been obtained by performing Rietveld quantitative phase analysis (QPA) on powder X-ray diffraction patterns and fitting the results with the Avrami model.

Furthermore, the thermal behavior of FAPI was studied by analyzing the TG/DSC (thermogravimetry/differential scanning calorimetry) curves, where only a three-step decomposition occurs in the temperature range explored. The DSC raw data after baseline correction and deconvolution were processed according to either the integral isoconversional method of Kissinger–Akahira–Sunose (KAS) or Šimon’s incremental isoconversional method. In the recent past, several studies were presented where similar methods were applied on dosage forms containing phosphomycin salts,^27^ transition metal ion complexes with salicylaldehydes,^28,29^ and real mixed plastics under pyrolytic conditions.^30^ The conversion dependences of activation energy were obtained for the three decomposition steps of FAPI and compared with the single activation energy value related to the nucleation and growth of the solid decomposition product PbI_2_.

Finally, Knudsen effusion techniques [Knudsen effusion mass spectrometry (KEMS) and Knudsen effusion mass loss (KEML)] were used with the twofold aim to investigate the gaseous phase evolved in close-to-equilibrium conditions during FAPI thermal decomposition and to estimate thermodynamic quantities associated to the process. The Gibbs energy changes of the two main decomposition channels identified by KEMS were determined by analyzing KEML data, and the first estimate of the enthalpy of formation of FAPI was thereafter derived.

## Experimental Section

### Material Preparation

Lead (II) nitrate Pb(NO_3_)_2_ (99%) and hydriodic acid HI 57% aqueous solution (<1.5% hypophosphorous acid as the stabilizer) were purchased from Alfa Aesar. Formamidinium iodide (FAI) was purchased from Sigma Aldrich. PbI_2_ had been previously prepared by reacting aqueous lead nitrate with aqueous hydriodic acid. Formamidinium lead iodide was prepared using a solid-state route. Stoichiometric amounts of FAI and PbI_2_ were carefully grinded together in an agate mortar until a homogeneous brown powder was obtained. The mixture was then transferred in a Pyrex weighing bottle and heated at 200 °C for 2 h, obtaining FAPI in the black phase. The completion of the reaction was checked by powder X-ray diffraction.

### TG/DTA/QMS

The composition of the evolved gas during the TG measurements was analyzed using a Netzsch STA 409 PC Luxx thermal analyzer working in TG-DTA mode coupled with a Balzers–Pfeiffer QMG 421 quadrupole mass spectrometer (QMS) in the *m*/*z* range of 0–128. The thermal analyzer outlet was connected to the inlet of the mass spectrometer to through a stainless-steel capillary heated at 150 °C to prevent moisture condensation. FAPI powder was placed in a sintered alumina crucible, and the scan was performed under a flowing Ar atmosphere (40 cm^3^/min @ STP, purity ≥99.9995%) with a scan rate of 10 K/min in the range RT–500 °C.

### TG/DSC Measurements for Isoconversional Kinetic Computations

Simultaneous TG/DSC measurements were carried out using a Stanton Redcroft apparatus (STA 625 model) equipped with two identical aluminum cylindrical crucibles (one for the sample and one, empty, for the reference). Sample sizes of 8–10 mg (precisely weighted) were heated in five single constant heating rate experiments at 2, 3, 4, 7, and 10 °C/min under a 50 mL/min Ar inert purging gas atmosphere. Calibration of temperature and heat flow was performed by comparing the melting temperature and the enthalpy of fusion of high-purity metals (indium and zinc in this study) with those recommended by the literature.^29,31^ Baseline correction of either TG or DSC signals has been performed. The former is addressed to avoid the initial mass gain due to the air buoyancy by performing a blank experiment under identical conditions, while that of the latter is carried out by considering by subtracting a sigmoidal shape of the blank curve to the sample curve in the temperature range of each DSC.

### X-ray Diffraction

Powder X-ray diffraction measurements were performed with a Panalytical X’Pert Pro MPD diffractometer (Cu Kα source, λ = 1.54184 Å) equipped with an ultra-fast X’Celerator RTMS detector. An Anton Paar XRK 900 was attached to the diffractometer to perform non-ambient temperature measurements. He gas flow (20 cm^3^/min @STP, purity 99.999%) was used as the protective atmosphere.

Kinetic data related to the decomposition of FAPI were obtained (temperature range 218–235 °C) by treating FAPI samples isothermally for 1 h at different temperatures, quenching them to room temperature, and acquiring their diffraction patterns. Eight temperatures were used, and eight diffraction patterns were acquired for each temperature after 1 h intervals. Quantitative phase analysis (QPA) of the diffraction patterns was performed using the MAUD Rietveld software package.^32^ The activation energy value for the reaction of formation of solid PbI_2_ from solid FAPI was obtained by the Arrhenius plot of the kinetic constant values of the FAPI decomposition reaction to PbI_2_ at each experimental temperature obtained by analyzing the data using the Johnson–Mehl–Avrami–Kolmogorov (JMAK) model.^33^

### KEMS and KEML

In the KEMS technique, the sample is placed in a capped cell with a small hole (1 mm diameter) done in the lid. The vapor effusing from the cell enters the electron impact ion source of a magnetic sector mass spectrometer where neutral species are positively ionized, accelerated, and focused in a beam that enters the magnetic analyzer region. Here, different ions are separated according to their mass/charge ratio. From the ion intensity of each species, the partial pressure of neutral precursors inside the cell can be calculated (see eq 10 below). Details of our KEMS apparatus are reported elsewhere.^34^ In the present experiments, the energy of the ionizing electron beam was set to 25 eV. Calibration of the instrument was performed by vaporizing pure cadmium. Temperature was measured by an iron–constantan thermocouple inserted in a hole done in the bottom of the Knudsen cell.

The KEML method is the classic version of the effusion-based techniques. The Knudsen cell containing the sample is placed inside a microthermobalance, and the weight loss rate is measured at a given temperature. The apparatus used in this work is a Ugine–Eyraud model Setaram B60 with homemade modifications.^35^ From the measured weight loss rate, the total pressure in the cell can be derived provided that the mean molecular weight of the vapor is known (for more details, see below the Results and Discussion sections).

## Theoretical Background of the Decomposition Kinetics

### JMAK Treatment of Temperature-Controlled X-ray diffraction (XRD) Data

The JMAK model is a purely geometrical model used to describe the volumetric transformation of one solid phase into another one by a nucleation and growth process.^33^

The JMAK equation used to describe the conversion of one solid phase into another solid phase B at a constant temperature is expressed by eq 1:
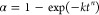
1where α is the volume fraction of the phase B, *k* is the kinetic constant, *t* is time, and *n* is the Avrami exponent^33^ (that in our case results equal to 1; see Supporting Information, Figures S1-S8).

The slope of the regression line of the ln *k* vs 1/*T* plot according to the well-known Arrhenius equation ln *k* = ln *A*–*E*_a_/*RT* (with *A* and *E*_a_ being the pre-exponential factor and the activation energy, respectively) provides a single value of *E*_a_, the sum of the contributions of nucleation and growth processes.

### Isoconversional Methods Applied by Processing DSC Data

A completely different approach to study the thermal decomposition kinetics of FAPI is based on two isoconversional methods, which are based on the fundamental kinetic equation (eq 2):
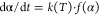
2where α is the degree of conversion (determined for each single step as the fractional area of the DSC peak related to the decomposition step), *T* is the absolute temperature, and *k* and *f* are the temperature and conversion functions, respectively. TG runs are usually performed under nonisothermal conditions at a constant heating rate (β = d*T*/d*t*), and after some rearrangements, dα/d*t* = d*T*/d*t*·dα/d*T* = β·dα/d*T*. If the temperature function is expressed according to the Arrhenius equation, eq 2 assumes the following form:
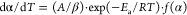
3and, after separating α and *T* variables, yields:
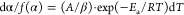
4

With integration of both hand-sides of eq 4, it can be written as:
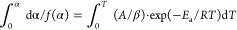
5where the temperature integral in eq 5 has no exact but approximate solutions. Several integral isoconversional methods can be considered depending on the approximation selected. One of the more reliable is the Kissinger–Akahira–Sunose (KAS) approach^36^ that enables to derive a single value of *E*_a_ at any given degree of conversion α from the slope of the regression line obtained by plotting ln(*β*/*T*^2^) vs 1/*T*.

The validity of the integral isoconversional methods is based on the assumption that the activation energy is practically constant (or at least its variation is negligible) over almost the whole range of α values. On the other hand, when a significant variation of the activation energy (more than 20% of the average value) is obtained, incremental isoconversional methods are a better choice. Among these methods, we selected that proposed by Šimon and co-workers,^37^ in which activation energy and pre-exponential factor values are obtained at each given value of α from the slope and intercept of the regression line displayed in eq 6:
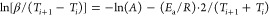
6where *A* and *E* are the Arrhenius pair related to the corresponding degree of conversion α.

To reach this goal, several TG, DTA, or DSC experiments (at least four to five) must be carried out at different heating rates. The results are usually expressed as *E*_a_ vs α plot.

## Results and Discussion

### Thermal Analysis Study

**Figure 1:**
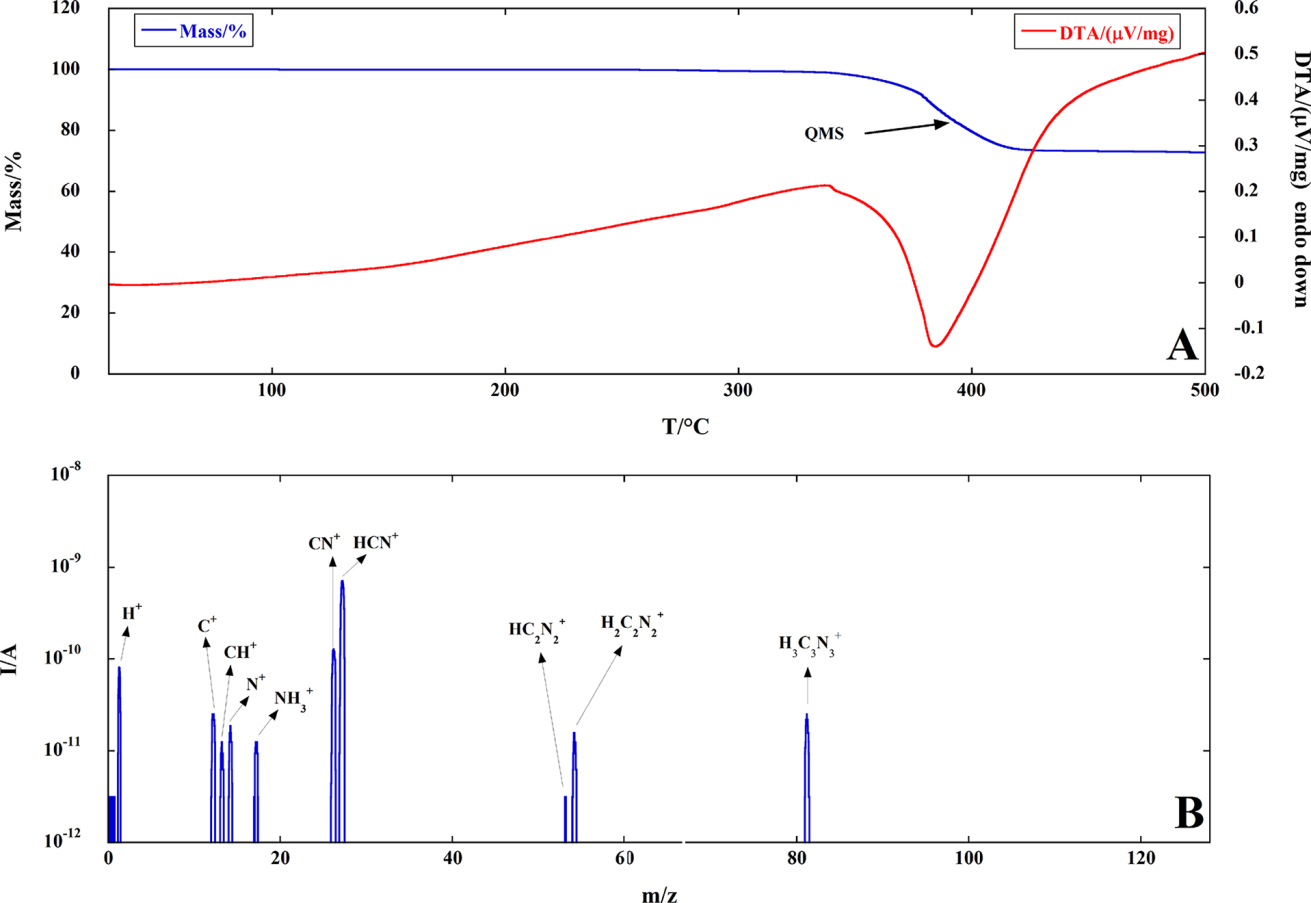
TG/DTA curve of FAPI under an Ar atmosphere at 10 °C/min (A) along with a background-subtracted QMS spectrum (B) representing the evolved gas analysis (EGA) at the point indicated by an arrow in panel A.

The thermal behavior of FAPI was studied by either coupling TG/DTA measurements with a quadrupole mass spectrometer (QMS) or comparing the TG/DTA curves with those recently published^38^ of tetramethylammonium lead iodide (TMAPI), measured under an inert atmosphere from ambient to 500 °C. The TG/DTA curves coupled with a background-subtracted QMS spectrum of FAPI are shown in Figure 1.

**Figure 2:**
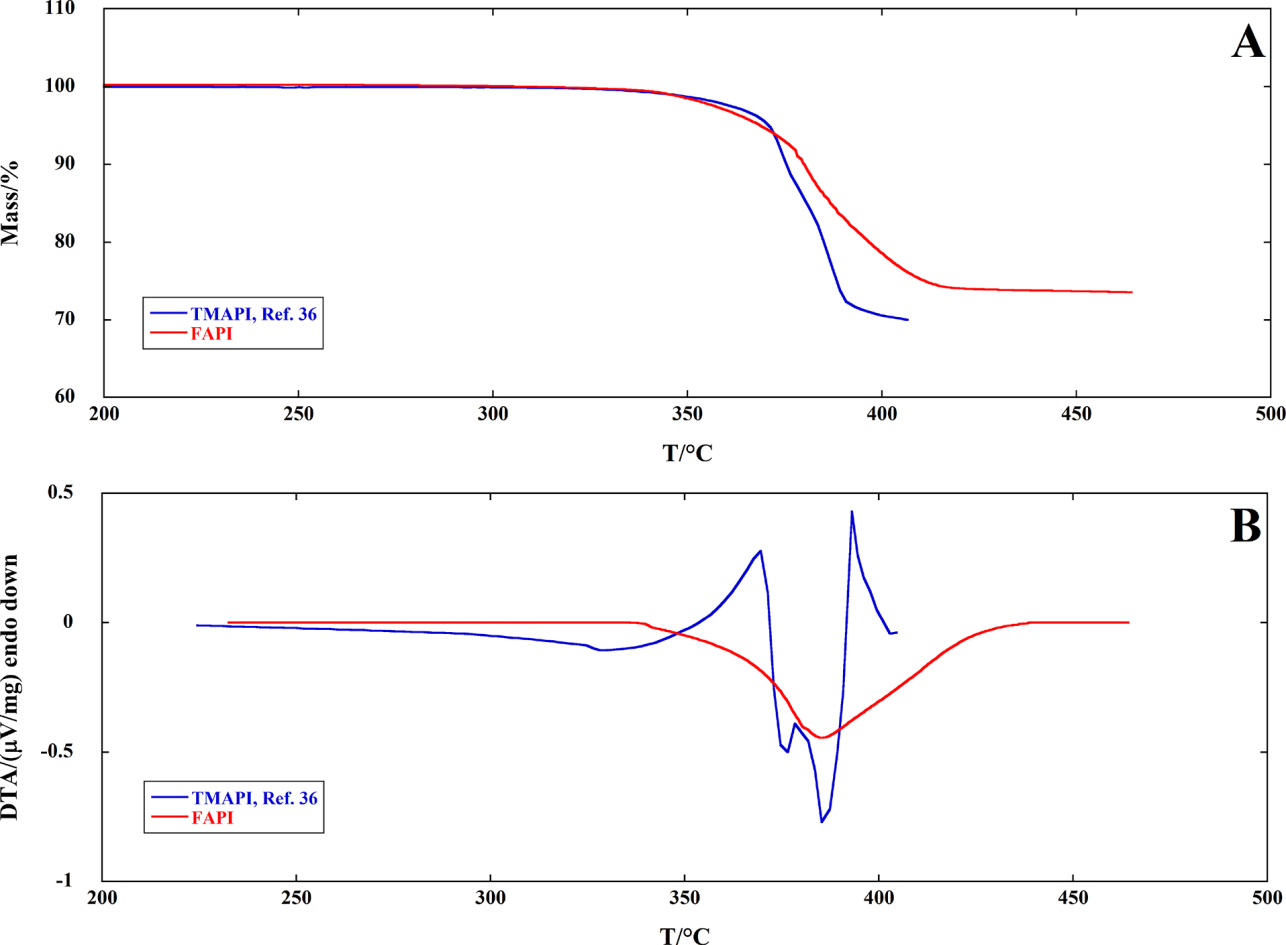
Comparison of TG (A) and DTA (B) curves of FAPI and TMAPI under flowing Ar at 10 °C/min. The endo/exothermic effects for the three-step decomposition of TMAPI are marked in panel B.

Furthermore, the TG/DTA curves of both lead iodide perovskites under identical conditions are shown in Figure 2, where no mass loss is recorded up to about 300 °C for both materials.

Both perovskites undergo a three-step decomposition, and the TG curves related to the first step of mass loss (up to about 370 °C corresponding to about 7% by mass) are almost superimposable. This result could lead to the conclusion that the first decomposition step of either FAPI or TMAPI may have the same reaction mechanisms in spite of the quite different shapes of both the corresponding DTA curves.

Starting from 370 °C, the decomposition of TMAPI occurs at lower temperatures with two sharper DTA peaks and ends at about 400 °C, while the second and third overlapping steps of FAPI decomposition take place in a wide temperature range, coming to an end at about 420–430 °C. The DTA curve of FAPI in Figure 2 is corrected using a sigmoidal-shaped baseline. The same correction was adopted for DSC curves performed at different heating rates and used for kinetic computation, as shown in the Supporting Information (Figure S9).

### Kinetic Analysis of FAPI Thermal Decomposition

**Figure 3:**
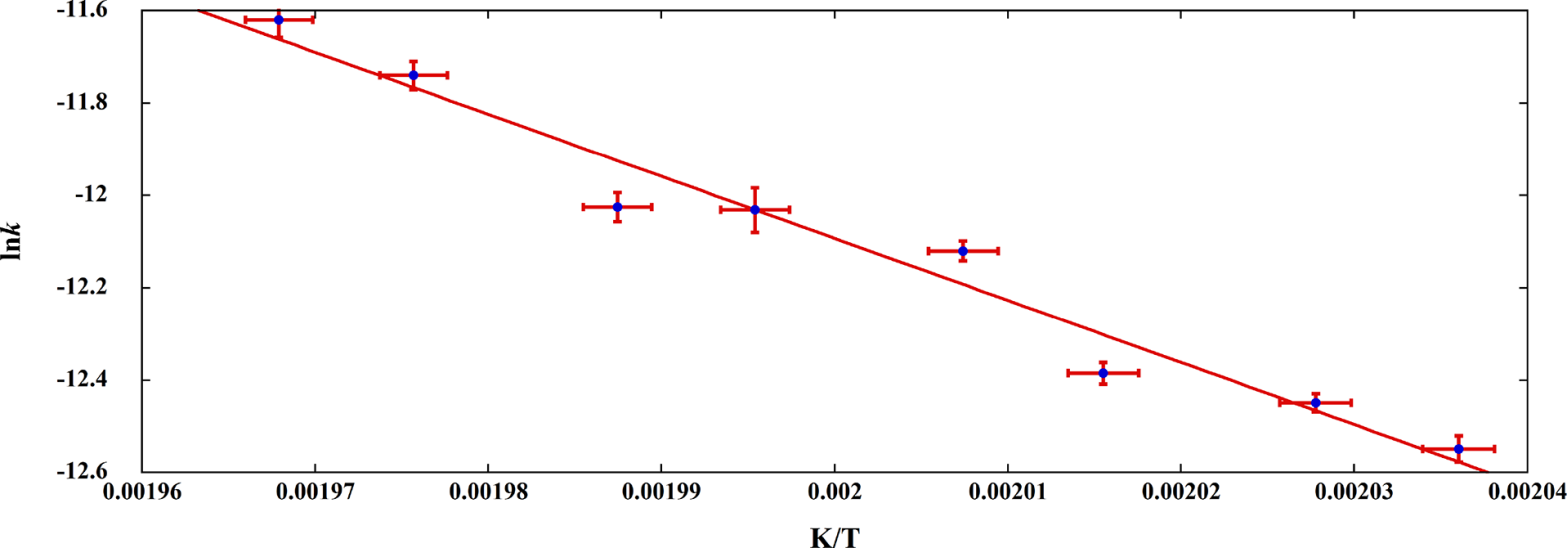
Arrhenius plot of the JMAK kinetic constants obtained by temperature-controlled XRD.

The kinetics of FAPI decomposition was studied by two experimentally independent approaches: the JMAK treatment of temperature-controlled XRD data and the isoconversional methods derived by treating experimental DSC data. By processing XRD data according to the JMAK method (Figure 3), from the slope of the Arrhenius plot ln*k* vs 1/*T* (with the *k* values obtained according to eq 1 at each fixed temperature), a value of *E*_a_ = 112 ± 9 kJ/mol is obtained.

**Figure 4:**
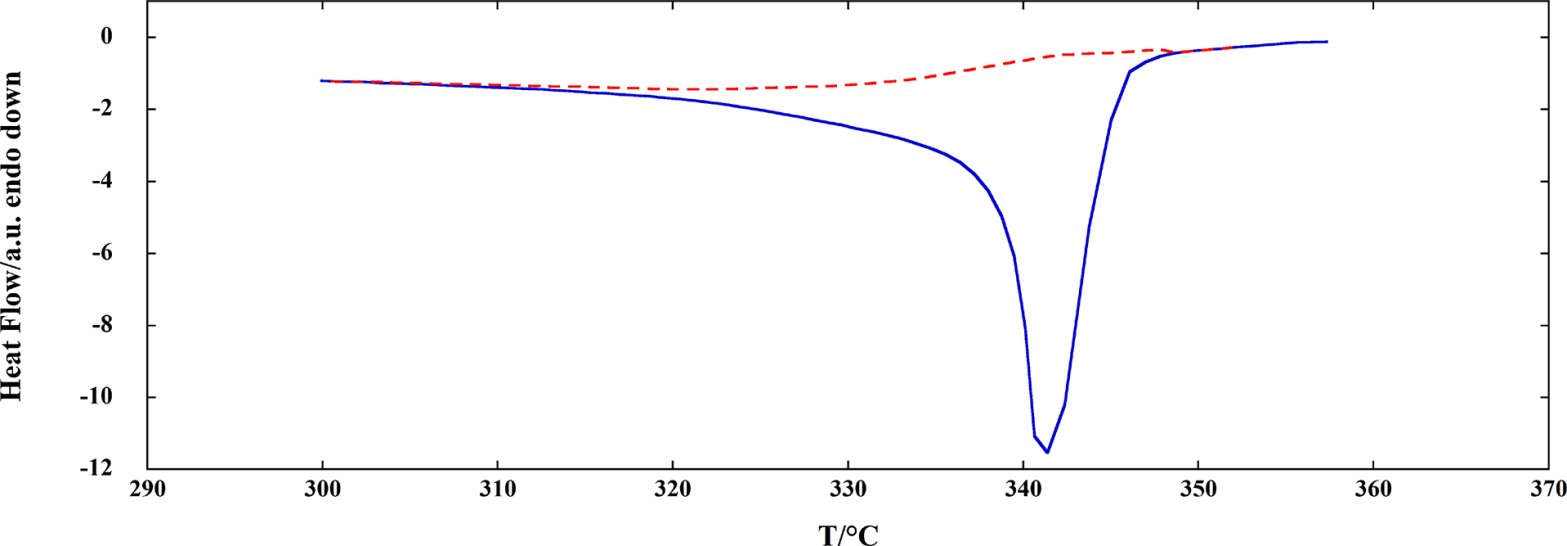
DSC curve of FAPI registered at 2 °C/min under flowing Ar (solid line) and peak baseline (dotted line).

With regard to DSC analysis, after baseline correction of the DSC peak related to the thermal decomposition of FAPI, a mathematical deconvolution into three Gaussian-shaped peaks was performed for each experiment at a constant heating rate. As an example, the DSC peak recorded at 2 °C/min is shown in Figure 4.

**Figure 5:**
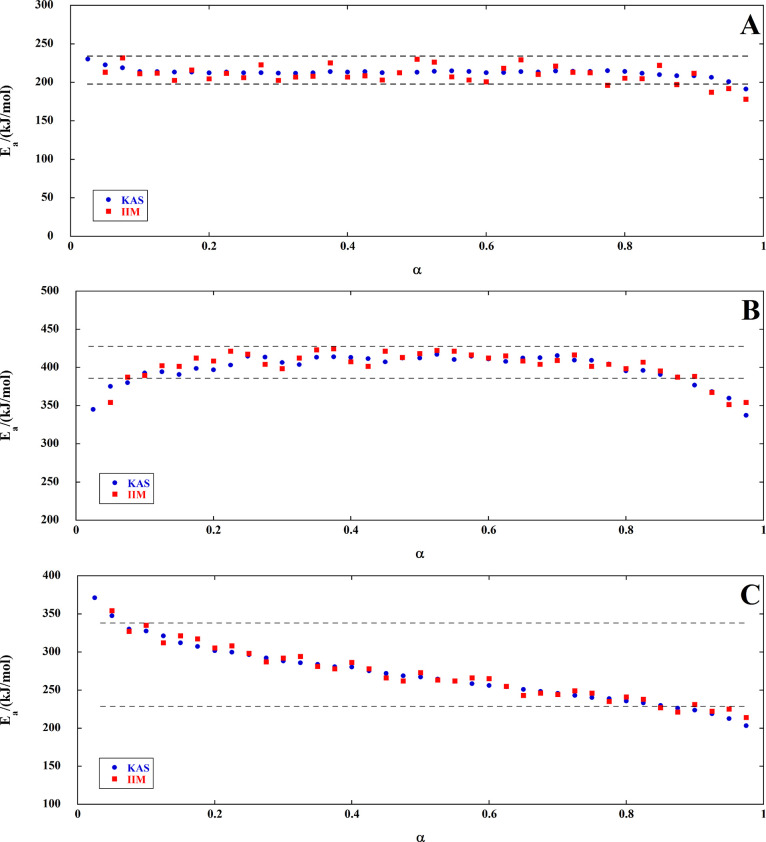
Conversion dependences of activation energy for the decomposition of FAPI. (A) First step, (B) second step, and (C) third step.

The results of both the KAS and incremental isoconversional methods are compared in the form of *E*_a_ vs α plots in Figure 5 (one plot for each of the three overlapping decomposition steps of FAPI with blue circles and red squares, respectively).

A very good agreement is observed between the activation energies of decomposition calculated by the KAS and those obtained by the incremental isoconversional methods for the three steps. As far as the first and the second steps are concerned, the activation energy is almost constant in the range 0.1 < α < 0.9, and mean *E*_a_ values can be considered: 205 ± 20 and 410 ± 20 kJ/mol, respectively, where the associated errors do not exceed the commonly estimated uncertainties. A decreasing trend of activation energy with the degree of conversion is observed for the third step (Figure 5C), with *E*_a_ that drops from 330 to about 220 kJ/mol. This remarkable change of activation energy makes impossible the use of the KAS method.^39^ Nevertheless, the *E*_a_ values calculated by the KAS method show an excellent agreement with those obtained with the incremental isoconversional method that does not require this a priori assumption.

### Decomposition Mechanisms for the Assessment of the Thermal Stability of FAPI

The assessment of thermal stability on the basis of the kinetic analysis of a multi-step decomposition process is a difficult task. To reach this goal, the first and the slowest steps are the most important ones. When comparing the mean activation energy of the first decomposition step calculated using the isoconversional methods and the one determined according to the JMAK model (205 ± 20 and 112 ± 9 kJ/mol, respectively), a remarkable difference is evident. To explain this apparent discrepancy, it is necessary to take into account the intrinsic differences of the information that can be gained from the different techniques and data treatment procedures.

Being based on a purely geometric nucleation-and-growth model, the JMAK approach enables obtaining an *E*_a_ value related to the rate of formation of the PbI_2_ solid product, monitored by the acquisition of XRD patterns at known time intervals (such rate might be in principle affected by possible slow decomposition steps of the organic cation). Subsequent steps involving the rearrangement of the cation fragments, the formation of gaseous end products, and their release are not expected to affect the calculated activation energy. On the contrary, the isoconversional methods are based on thermal analysis data and provide a set of *E*_a_ values that are expected to encompass the effect of the rearrangement/migration/ release of the gaseous species.

The deviation observed between the activation energy value obtained by JMAK and the mean value derived by the isoconversional methods supports the hypothesis of a multi-step process, in which the desorption of the gaseous products does not occur simultaneously with the nucleation and growth of PbI_2_, but these last processes occur before the gas release. If the desorption of the gaseous species could take place simultaneously or before the nucleation and growth of PbI_2_, then the two energy barriers determined by JMAK and isoconversional procedures would agree (within the experimental uncertainties) since the gas release would be a prerequisite for the formation of PbI_2_. In addition, the hypothesis of a multi-step process in which the nucleation and growth of PbI_2_ are followed by the release of gas is also supported by the complexity of the gaseous phase produced during the decomposition of FAPI. The mass spectrum recorded (with maximum ion current intensities due to evolved gases) during a TG/DTA measurement (Figure 1B) agrees with data already reported in literature,^40^ i.e., the production of sym-triazine H_3_C_3_N_3_, (HCN)_2_, HCN (these last two could be fragments of sym-triazine), and NH_3_. The production of one sym-triazine or (HCN)_2_ molecule requires the reaction of more than one formamidinium moiety, and this is much more probable to occur in the condensed phase, where their concentration is higher, than in a diluted gaseous phase. Though invisible by XRD, the formation of these species may have a profound effect in the overall value of the *E*_a_, requiring the breaking of a stable C–N bond,^38^ and in fact, their contribution is clearly visible in the activation energy value obtained by the KAS analysis, which is the sum of all the contributions. In the next section, the analysis of the gas phase performed under effusion (i.e., closer-to-equilibrium) conditions is presented.

### KEMS Results: Composition of the Gas Phase and Decomposition Pathways

**Figure 6:**
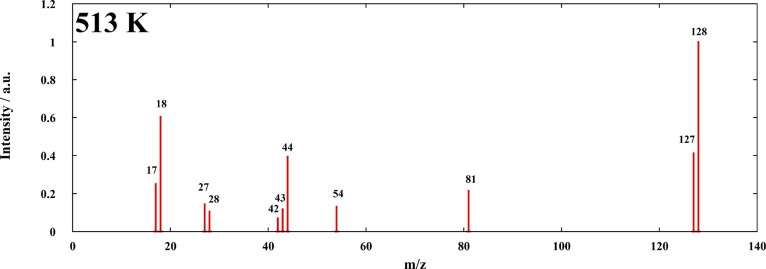
Background-subtracted mass spectrum of the vapor produced by FAPI under effusion conditions.

To investigate the species released into the gas phase from FAPI upon heating, Knudsen effusion mass spectrometry experiments were carried out in the 476–519 K temperature range. In this technique, the vapors effusing from a Knudsen cell are analyzed mass spectrometrically. The contribution to the signal coming from background species outside the cell is subtracted with the help of a movable shutter placed between the cell and the ion source. A typical background-subtracted mass spectrum obtained from the FAPI vapor is reported in Figure 6.

A reasonable assignment of the most intense peaks to the corresponding neutral precursors is as follows (fragment ions are included): formamidine, CN_2_H_4_ (peaks at *m*/*z* = 42, 43, and 44); hydrogen iodide, HI (128 and 127); triazine, C_3_H_3_N_3_ (81, 54, and 27); and ammonia, NH_3_ (17). Finally, a rather intense peak at *m*/*z* = 18 is observed, which may be assigned to water. This peak decreased significantly as the experiment proceeded. Note that the presence of H_2_O can give a contribution to the *m*/*z* = 17 intensity due to the OH^+^ fragment, causing a small error in the NH_3_ measurement.

In addition to being triazine fragments, the peaks at *m*/*z* = 27 and 54 are probably due to HCN and (to a lesser extent) H_2_C_2_N_2_ neutral precursors. However, the reference mass spectrum of triazine^41^ displays intense peaks at these *m*/*z* values (and at *m*/*z* = 28 too) due to fragmentation under electron impact, and our data do not permit to distinguish the two contributions. For this reason, in the following analysis of partial pressures and thermodynamic properties, we preferred not to include these species. The release of HCN from the decomposition of FAPI or FAPI iodide precursor was indeed reported in previous papers,^40,42,43^ although discrepancies exist with regard to the temperature ranges where the evolution of this species dominates on other decomposition products (see below).

Based on the mass spectrum in Figure 6 and the above discussion, we recognize the presence in the gas phase of the formamidine (CN_2_H_4_), hydrogen iodide (HI), triazine (C_3_H_3_N_3_), ammonia (NH_3_), and hydrogen cyanide (HCN) species. As discussed in the previous section, the only solid decomposition product is PbI_2_, as confirmed by the XRD analysis of the vaporization residues of KEMS experiments.

It is then reasonable to conclude that in the temperature range covered by KEMS experiments (476–519 K), the main decomposition reactions simultaneously underwent by FAPI are the following:
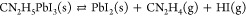
7
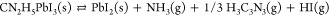
8

As reported above, the KEMS spectra additionally confirm the presence of HCN released by the process:
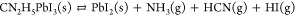
9but further work is necessary to distinguish between the contributions to the *m*/*z* = 27 signal coming from the triazine fragmentation and neutral HCN(g). Note that while process 7 involves only a proton exchange among the organic cation and the iodide anion, processes 8 and 9 require the rupture of the C–N bond, which can imply a high activation energy.^38^

The occurrence of the above reactions was suggested in previously published papers on the basis of quadrupole mass spectrometry,^40^ FTIR spectroscopy,^42^ and GC–MS^43^ experiments. According to the results of ref (40), the release of triazine becomes significant above 95 °C, with HCN and formamidine being the main decomposition products at lower temperatures (close to the conditions of solar cell applications). However, FTIR results^42^ would suggest HCN to become dominant only above 360 °C. Furthermore, FTIR spectra do not show evidence of the release of formamidine, which was instead observed in ref (40) and is now confirmed by our KEMS spectra (peaks at *m/z* = 42, 43, and 44). Note also that the formamidine peaks are not present in the TG-QMS spectrum shown in Figure 1. However, a comparison between the results obtained in the various studies mentioned above should be done with caution because the experimental conditions are not the same. In particular, no work was previously done under effusion conditions. The applied experimental conditions are of particular importance in cases such as this one, where the release of gas from the decomposing solid may be slow (see previous sections), especially for the gaseous species whose formation involves a relatively complex sequence of bond breaking/formation.

To attempt an estimate of the relative importance of the two decomposition channels, the partial pressures *p_i_* of the gaseous species are to be estimated by the KEMS equation:
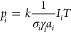
10where *k* is an instrumental constant; *I_i_* the measured ion current; and σ_*i*_, γ_*i*_, and *a_i_* are the electron impact ionization cross section, the multiplier gain, and the isotope abundance of the species *i*. To this end, the σ value thus has to be known. While this is the case for HI and NH_3_ (6.5 and 3.0 Å^2^, respectively^44,45^), the remaining values can be roughly estimated by the element additivity rule, which implies possible inaccuracies. Within the limits of this approximation, the partial pressures reported in Table S1 (Supplementary Information) were obtained. It is interesting to note that the mean values of the formamidine/ammonia and formamidine/triazine pressure ratios (0.9 and 3.8, respectively) suggest that the contribution of processes 1 and 2 is of similar extent, with no process clearly dominating (under effusion conditions) in the explored temperature range.

### KEML Results: Total Decomposition Pressure and Thermodynamic Evaluation

To obtain more accurate pressure values and to estimate the thermodynamic properties of the decomposition reactions, extensive experiments were carried out by the Knudsen effusion mass loss in the 398–487 K temperature range. In this technique, the total pressure *P* is determined by measuring the mass loss rate Δ*m*/Δ*t* provided that the mean molecular weight of the vapor species is independently known:^38^
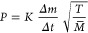
11

where *K* is an instrumental constant and *M̅* is the average molecular weight given by:
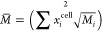
12

Since the effusion rates are inversely proportional to the square root of the mass, for a given decomposition process, the relative abundance of heavier species in the cell (*x*_*i*_^cell^ in eq 12) is higher than the stoichiometric one, which is instead maintained in the effusate.^40^ If, as in this case, the decomposition occurs by more than one process, the composition of the vapor phase depends on the relative importance of the various decomposition pathways, and as a consequence, *M̅* is expected to be temperature dependent. Since the mean molecular weight of the species released in reactions 7, 8, and 9 are, respectively, 92, 87, and 69 amu, a mean value within this range is expected as the result of their simultaneous occurrence.

**Figure 7:**
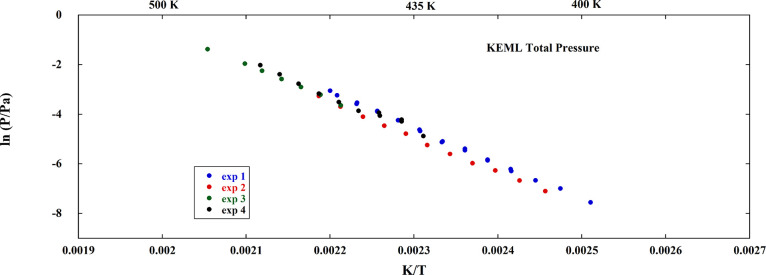
Total pressure of the gas phase released by FAPI as measured by KEML (four experimental runs), estimated by combining KEML and KEMS experiments (see text).

Within the limits of accuracy discussed above, the mole fractions to be used in eq 12 may be estimated by the KEMS partial pressures (see previous section). The calculated mole fractions are reported in Figure S2. Although the values are rather scattered, the mean value of 79 amu is consistent with the above-mentioned range expected for reactions 7–9. Indeed, in view of the uncertainties in the cross sections and fragmentation effects, this value is to be considered consistent with all three of these reactions. From Figure S2, a weak increasing trend with temperature may also be seen, suggesting that the importance of reaction 7 could increase with temperature. This evidence would support what has been reported in ref (40). However, more extensive KEMS measurements are necessary to give this conclusion a sound experimental basis. It should be noted that the temperature range covered by KEML measurements (398–487 K) is barely overlapped with that used in KEMS measurements (476–513 K), which adds uncertainty to the use of KEMS mole fractions in eq 12. For this reason, in calculating the total pressures by eq 11, we preferred to use the value *M̅* = 90 amu, intermediate between those corresponding to reactions 7 and 8 that were selected on the basis of KEMS spectra. The total pressures resulting from the four KEML runs are reported in Table S2 and are displayed in Figure 7. The uncertainty on these values is estimated at around 30%.

**Figure 8:**
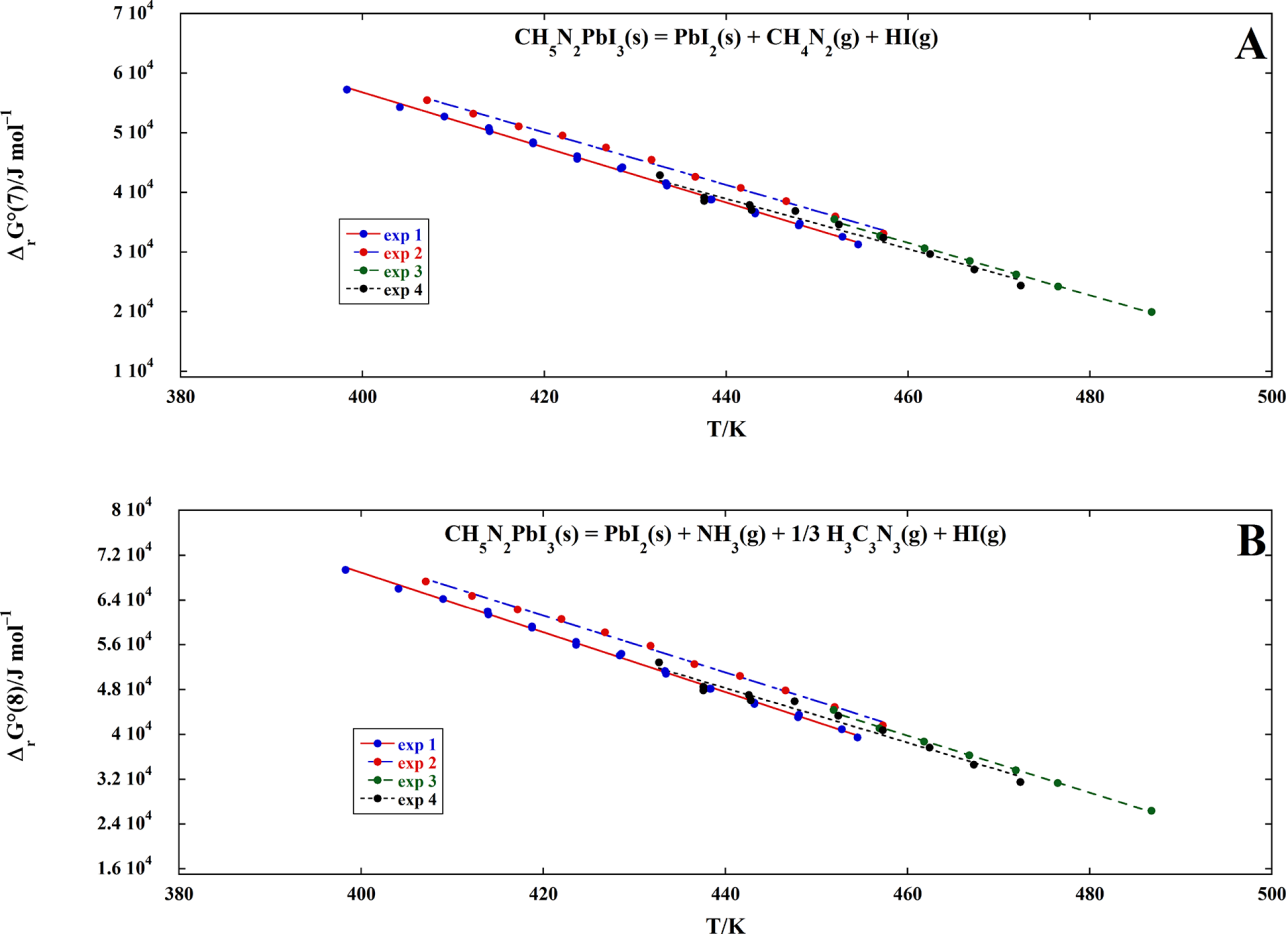
Standard Gibbs energy change (Δ_r_*G*°) of the decomposition reactions 7 (A) and 8 (B) as a function of temperature, estimated by combining KEML and KEMS experiments (see text).

Once the total pressure *P* in the cell is known from KEML measurements, it is possible to estimate the partial pressures by using KEMS mole fractions. To this end, we used the mole fractions derived from the KEMS data at the lowest temperature, neglecting any temperature dependence of the vapor composition in the KEML range (a rather crude approximation). Finally, the equilibrium constants and the standard Gibbs energy change (Δ_r_*G*°) of the decomposition reactions can be in turn evaluated. In Figure 8A,B, we report the Δ_r_*G*° of reactions 7 and 8 calculated from the four KEML experiments. In view of the acceptable agreement between the different runs, we propose the following equations for the Δ_r_*G*° (in kJ/mol) of the two reactions:
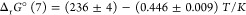
13
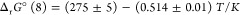
14calculated as the mean of the four regression lines, weighted by the number of data points. In eqs 13–14, the constant term and the temperature coefficient can be seen, respectively, as the Δ_r_*H*° and the Δ_r_*S*° at the average temperature (440 K).

In assessing our analysis, it should be emphasized that the kinetic results discussed in the previous sections cast a shadow on the attainment of thermodynamic equilibrium even under effusion conditions, especially for process 8 that involves the breaking of a C–N bond. In this connection, we note that by combining the enthalpies of reaction from eqs 13–14 with the enthalpies of formation of sym-H_3_C_3_N_3_(g) and HCN(g) (225.9 and 135.1 kJ/mol, respectively, at 298 K) and the pertinent heat contents to shift the values to 440 K,^46^ it is possible to derive the enthalpy change for the gas-phase decomposition of formamidine
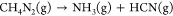
15

The value so obtained, Δ_r_*H*° (440 K) = 61 kJ/mol, is significantly higher than the *ab initio* value^47^ Δ_r_*H*°(298 K) = 22.3 kJ/mol (since the heat contents of formamidine are apparently not known, a comparison at the same temperature cannot be done, but this is expected to affect the values to a small extent). Although the theoretical value could be inaccurate to some extent, this discrepancy could indicate that a true thermodynamic equilibrium was not attained during effusion experiments so that the pressures measured for process 8 are underestimated and the enthalpy change is overestimated due to kinetic factors. However, it is likely that process 7 did attain thermodynamic equilibrium under the used conditions. From eq 13, it is then possible to propose a rough estimate of the enthalpy of formation of FAPI from elements by a simple thermochemical cycle using the enthalpy of formation values of PbI_2_(s), HI(g), HCN(g), and NH_3_(g), which are known from the literature, and the above-mentioned theoretical result for reaction 15. The resulting value is Δ_f_*H*°(FAPI, 440 K) = −353.1 ± 10 kJ/mol, where the uncertainty includes the heat capacity change neglected in reaction 15. To the best of our knowledge, no calorimetric determination of the formation enthalpy has been previously reported for this perovskite.

The comparison of the total decomposition vapor pressure of FAPI with the corresponding values obtained for MAPI and TMAPI^38^ shows that FAPI is sensibly more stable than MAPI, in agreement with the higher basicity of formamidine (p*K*_b_ = 2.5)^48^ compared to methylamine (p*K*_b_ = 3.36),^49^ though not as stable as TMAPI in which no acidic protons are present. This observation is in agreement with our previous work; i.e., more basic amines produce more stable perovskites by inhibiting acid–base equilibria responsible for decomposition reactions.^38,50^

## Conclusions

The kinetic and thermodynamic stability of formamidinium lead iodide FAPI has been experimentally studied by using a multi-technique approach. Compared to the prototypical perovskite MAPI, FAPI possesses a more complicated decomposition behavior, as evidenced by mass spectrometry measurements as well as by the discrepancies between the JMAK and isoconversional activation energy values for the decomposition process.

The thermodynamic analysis allowed us to obtain standard thermodynamic function values that are fundamental to predict the stability of FAPI in operative conditions in real devices.

The stability of FAPI results to be intermediate between MAPI (lower) and TMAPI (higher), making it more promising than MAPI for stable photovoltaic devices.

## Supporting Information Available

Additional thermodynamic results, JMAK plots, and deconvolution of FAPI decomposition DSC peak at different heating rates (PDF)
