# Supplementary material for: Thermodynamic and Kinetic Aspects of Formamidinium Lead Iodide Thermal Decomposition
Source: J Phys Chem C Nanomater Interfaces. 2021 Sep 30;125(40):21851–61. doi: 10.1021/acs.jpcc.1c06729 (PMC8521522; doi:10.1021/acs.jpcc.1c06729)
Supplement: Supplementary file 1 — jp1c06729_si_001.pdf [file jp1c06729_si_001.pdf]

**Supporting Information**

# Thermodynamic and Kinetic Aspects of Formamidinium Lead Iodide Thermal Decomposition

Alessio Luongo[1], Bruno Brunetti[2], Stefano Vecchio Ciprioti[3*], Andrea Ciccioli[1*] and Alessandro Latini[1*]

[1] Dipartimento di Chimica, Sapienza Università di Roma, Piazzale Aldo Moro 5, 00185 Roma, Italy

[2] Consiglio Nazionale delle Ricerche - Istituto per lo Studio dei Materiali Nanostrutturati, c/o Dipartimento di Chimica, Sapienza Università di Roma, Piazzale Aldo Moro 5, 00185 Roma, Italy

[3]Dipartimento S.B.A.I, Sapienza Università di Roma, Via del Castro Laurenziano 7, 00161 Roma, Italy

*corresponding authors.

**Table S1**. Partial pressures of the main gaseous species estimated by KEMS measurements.

| | T/K | $pNH_3$/ Pa | $pCH_4N_2$/ Pa | $p(HCN)_3$/ Pa | pHI/ Pa | $p_{TOT}$/ Pa |
|---|---|---|---|---|---|---|
| ***Run 1*** | 476 | $8,75*10^{-2}$ | $7,62*10^{-2}$ | $6,60*10^{-8}$ | $1,47*10^{-1}$ | $3,17*10^{-1}$ |
| | 488 | $1,79*10^{-1}$ | $1,46*10^{-1}$ | $2,25*10^{-2}$ | $3,95*10^{-1}$ | $7,42*10^{-1}$ |
| | 513 | $2,98*10^{-1}$ | $4,62*10^{-1}$ | $1,16*10^{-1}$ | $1,50*10^{0}$ | $2.37*10^{0}$ |
| ***Run 2*** | 513 | $6,75*10^{-1}$ | $5,85*10^{-1}$ | $1,99*10^{-1}$ | $1,86*10^{0}$ | $3,31*10^{0}$ |
| | 502 | $2,30*10^{-1}$ | $2,56*10^{-1}$ | $8,35*10^{-2}$ | $7,56*10^{-1}$ | $1,32*10^{0}$ |
| | 488 | $9,46*10^{-2}$ | $1,17*10^{-1}$ | $3,15*10^{-2}$ | $2,95*10^{-1}$ | $5,38*10^{-1}$ |
| ***Run 3*** | 505 | $5,87*10^{-1}$ | $3,23*10^{-1}$ | $2,66*10^{-1}$ | $1,61*10^{0}$ | $2,78*10^{0}$ |
| | 480 | $1,27*10^{-1}$ | $7,68*10^{-2}$ | $7,32*10^{-2}$ | $3,33*10^{-1}$ | $6,10*10^{-1}$ |
| | 519 | $7,63*10^{-1}$ | $3,27*10^{-1}$ | $3,67*10^{-1}$ | $2,10*10^{0}$ | $3,55*10^{0}$ |
| | 497 | $2,79*10^{-1}$ | $3,13*10^{-1}$ | $1,17*10^{-1}$ | $6,72*10^{-1}$ | $1,38*10^{0}$ |

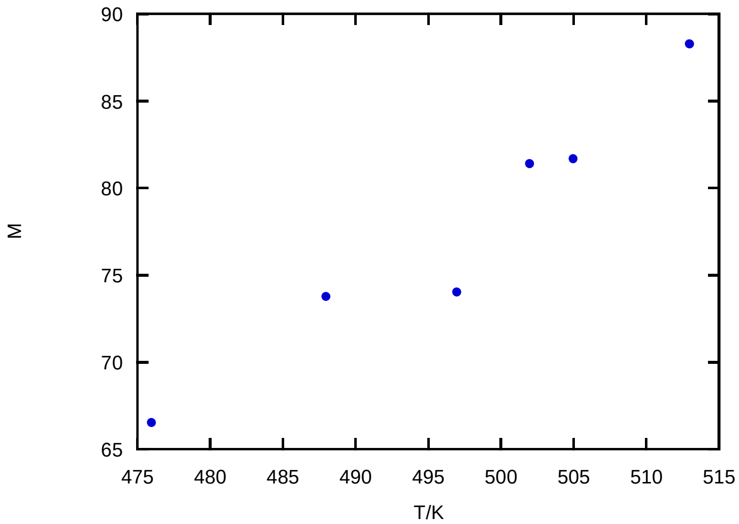


**Fig. S2**. Mean molecular weight calculated from KEMS spectra.

**Table S2**. Total pressures measured by KEML[a]

| **Experiment** | **T/K** | **P/Pa** |
|---|---|---|
| Exp 1 | 452.8 | 0.040 |
| | 448.0 | 0.029 |
| | 443.2 | 0.021 |
| | 438.3 | 0.015 |
| | 433.5 | 0.0099 |
| | 428.4 | 0.0062 |
| | 423.6 | 0.0046 |
| | 418.8 | 0.0028 |
| | 414.0 | 0.0020 |
| | 454.5 | 0.048 |
| | 448.1 | 0.028 |
| | 443.2 | 0.021 |
| | 438.4 | 0.015 |
| | 433.4 | 0.0094 |
| | 428.6 | 0.0060 |
| | 423.6 | 0.0043 |
| | 418.8 | 0.0029 |
| | 413.9 | 0.0019 |
| | 409.0 | 0.0013 |
| | 404.1 | 0.00092 |
| | 398.3 | 0.00053 |
| | | |
| Exp 2 | 457.3 | 0.039 |
| | 452.0 | 0.025 |
| | 446.6 | 0.017 |
| | 441.6 | 0.012 |
| | 436.6 | 0.0085 |
| | 431.8 | 0.0053 |
| | 426.8 | 0.0037 |
| | 422.0 | 0.0026 |
| | 417.2 | 0.0019 |
| | 412.2 | 0.0013 |
| | 407.1 | 0.00083 |
| | | |
| Exp 3 | 486.8 | 0.25 |
| | 476.5 | 0.14 |
| | 471.9 | 0.11 |
| | 466.8 | 0.076 |
| | 461.8 | 0.056 |
| | 456.9 | 0.041 |
| | 451.9 | 0.027 |
| | | |
| Exp 4 | 442.8 | 0.020 |
| | 437.6 | 0.015 |
| | 432.7 | 0.0077 |
| | 472.4 | 0.13 |

| | | |
|---|---|---|
| | 467.3 | 0.092 |
| | 462.4 | 0.063 |
| | 457.3 | 0.042 |
| | 452.4 | 0.030 |
| | 447.6 | 0.021 |
| | 442.6 | 0.017 |
| | 437.6 | 0.014 |

[a] In calculating pressures from the mass loss rate, a mean molecular weight of 90 u was assumed (see text)

**Figures S1-S8**. JMAK plots in the temperature range 218-235 °C

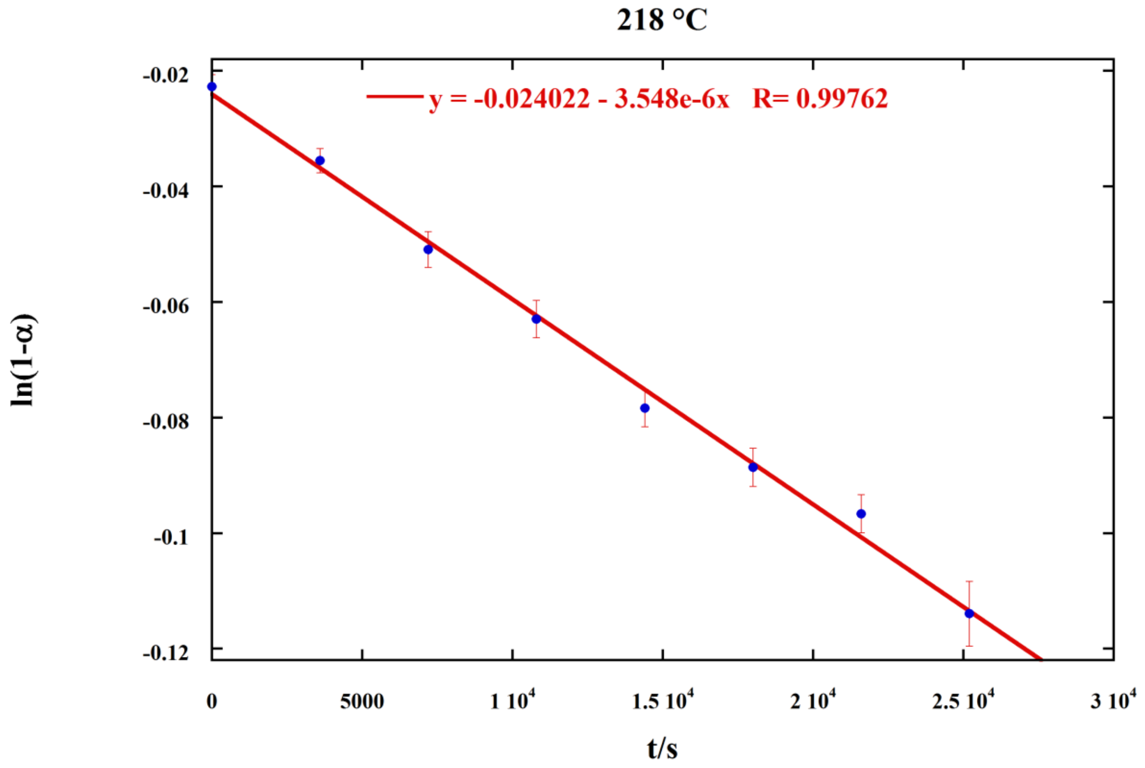


**Fig. S1**

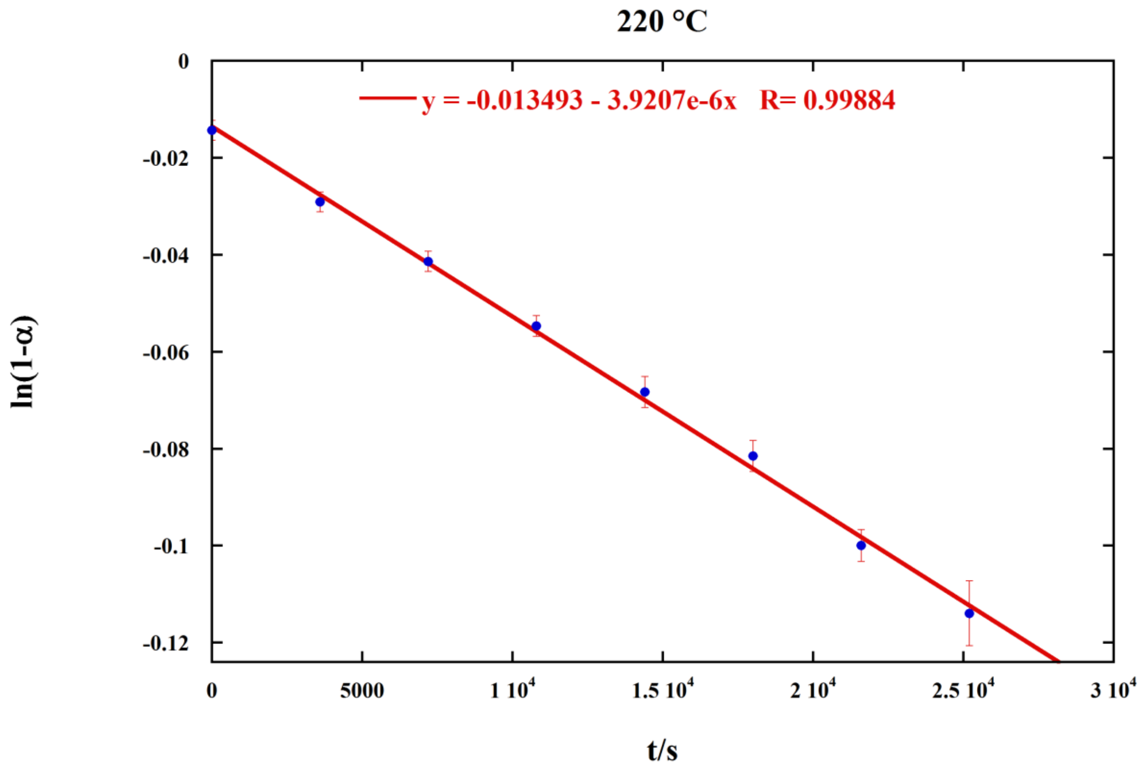


**Fig. S2**

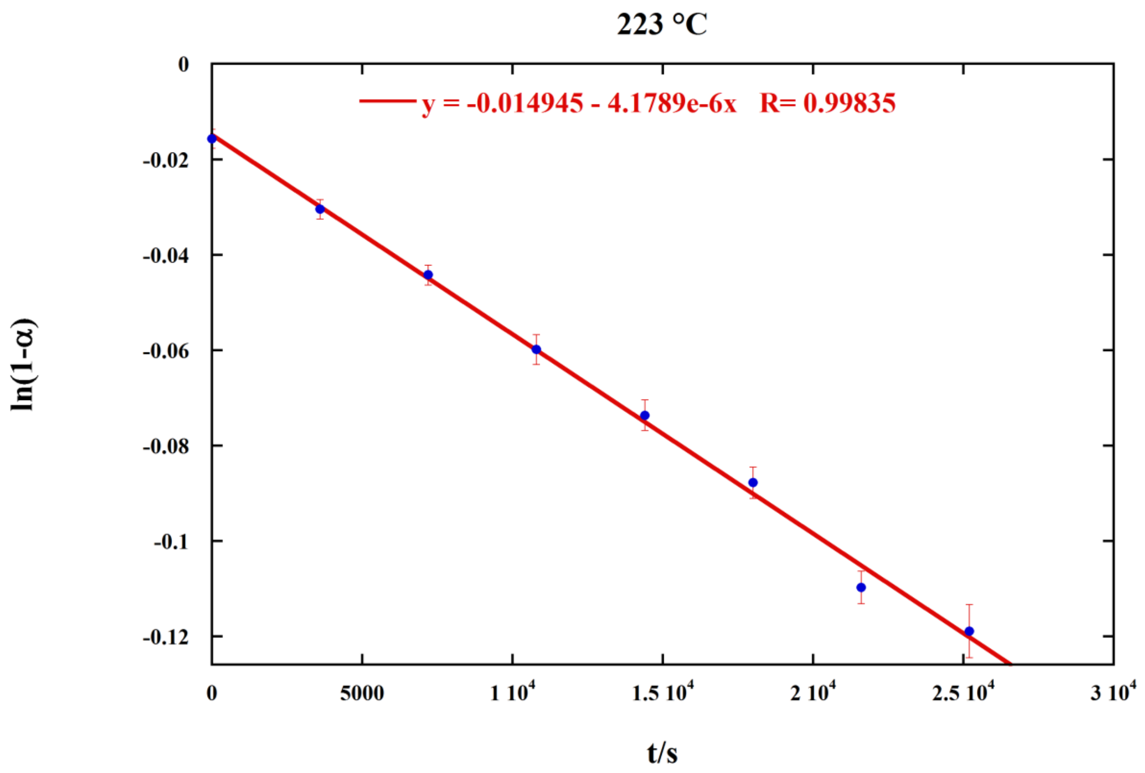
223 °C
y = -0.014945 - 4.1789e-6x   R= 0.99835
ln(1-α)
t/s
0
-0.02
-0.04
-0.06
-0.08
-0.1
-0.12
0
5000
1 10^4
1.5 10^4
2 10^4
2.5 10^4
3 10^4

**Fig. S3**

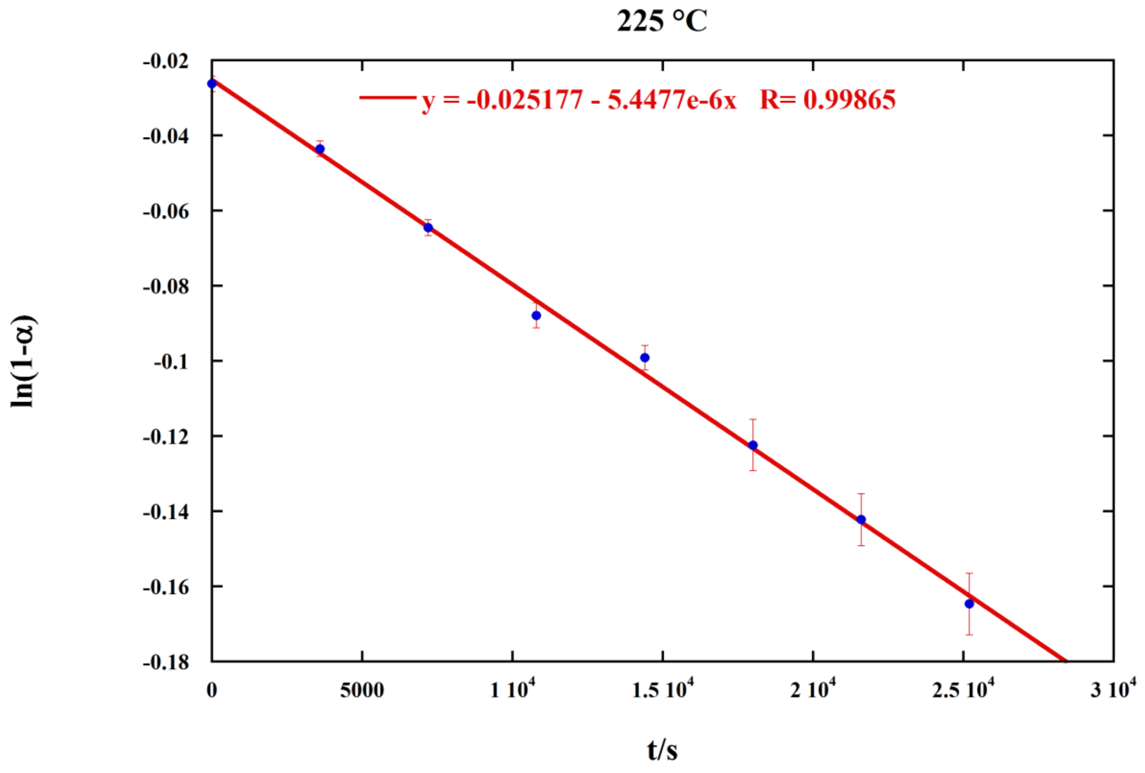
225 °C
y = -0.025177 - 5.4477e-6x   R= 0.99865
ln(1-α)
t/s
-0.02
-0.04
-0.06
-0.08
-0.1
-0.12
-0.14
-0.16
-0.18
0
5000
1 10^4
1.5 10^4
2 10^4
2.5 10^4
3 10^4

**Fig. S4**

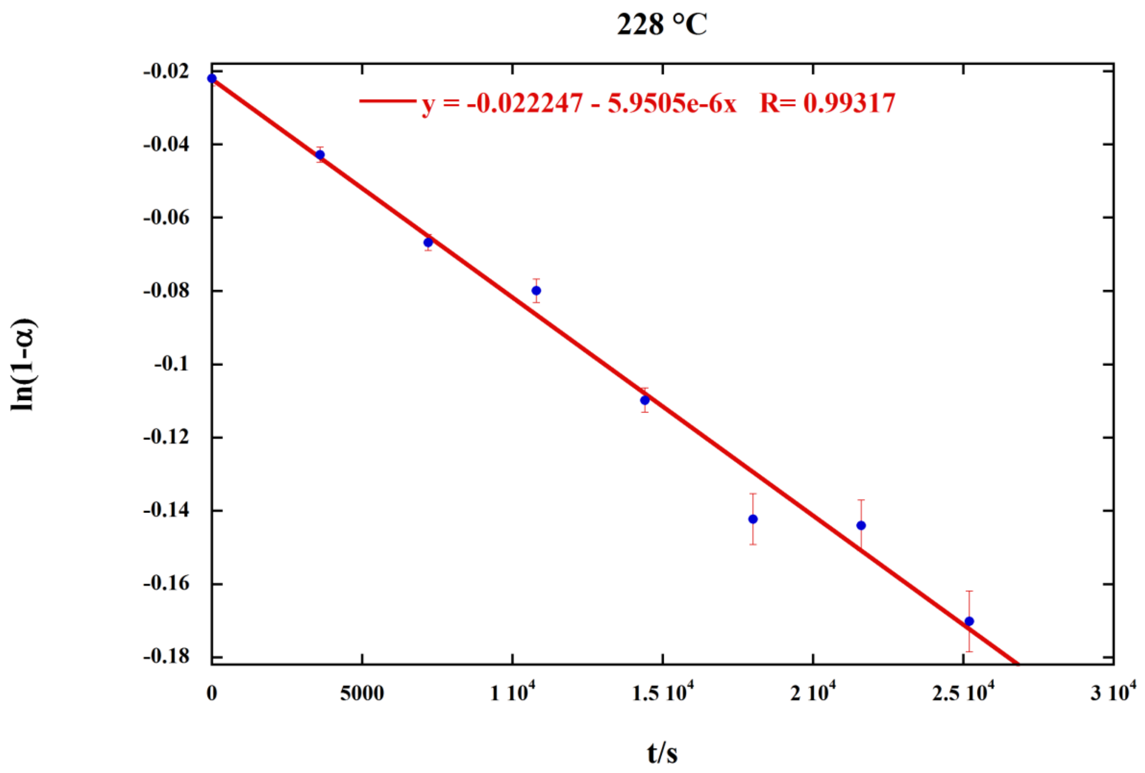

228 °C
y = -0.022247 - 5.9505e-6x R= 0.99317
-0.02
-0.04
-0.06
-0.08
-0.1
-0.12
-0.14
-0.16
-0.18
ln(1-α)
0
5000
1 10^4
1.5 10^4
2 10^4
2.5 10^4
3 10^4
t/s


**Fig. S5**

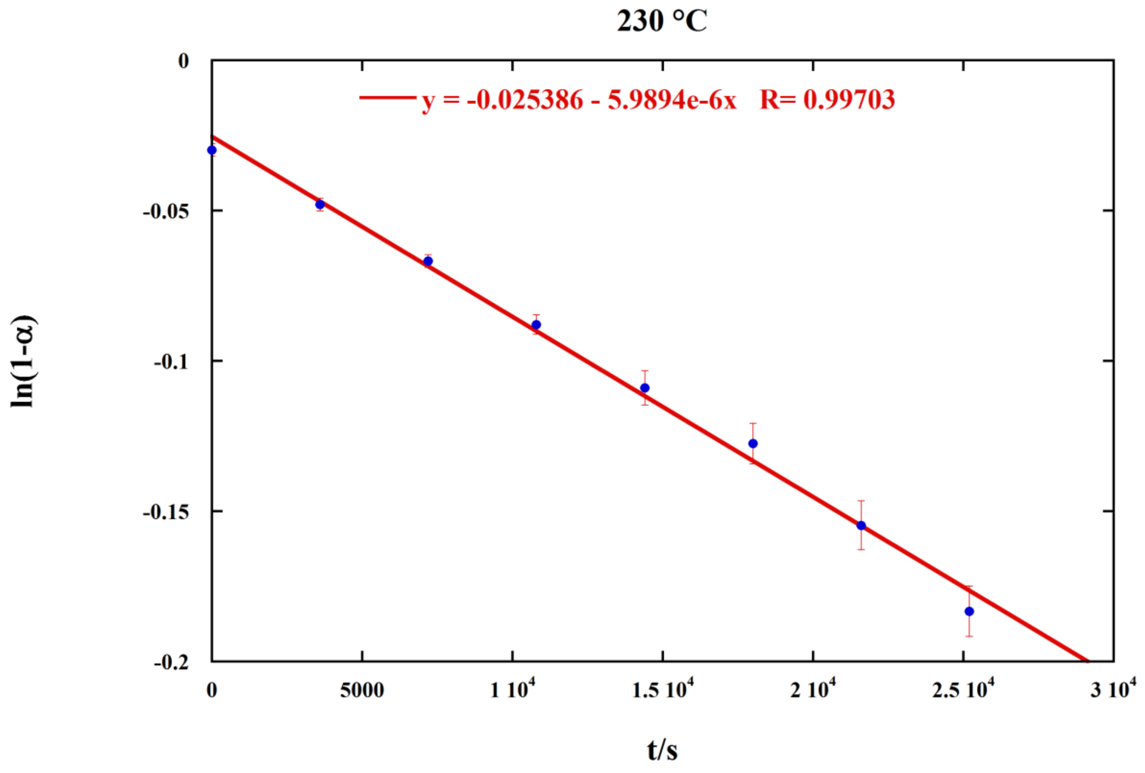

230 °C
y = -0.025386 - 5.9894e-6x R= 0.99703
0
-0.05
-0.1
-0.15
-0.2
ln(1-α)
0
5000
1 10^4
1.5 10^4
2 10^4
2.5 10^4
3 10^4
t/s


**Fig. S6**

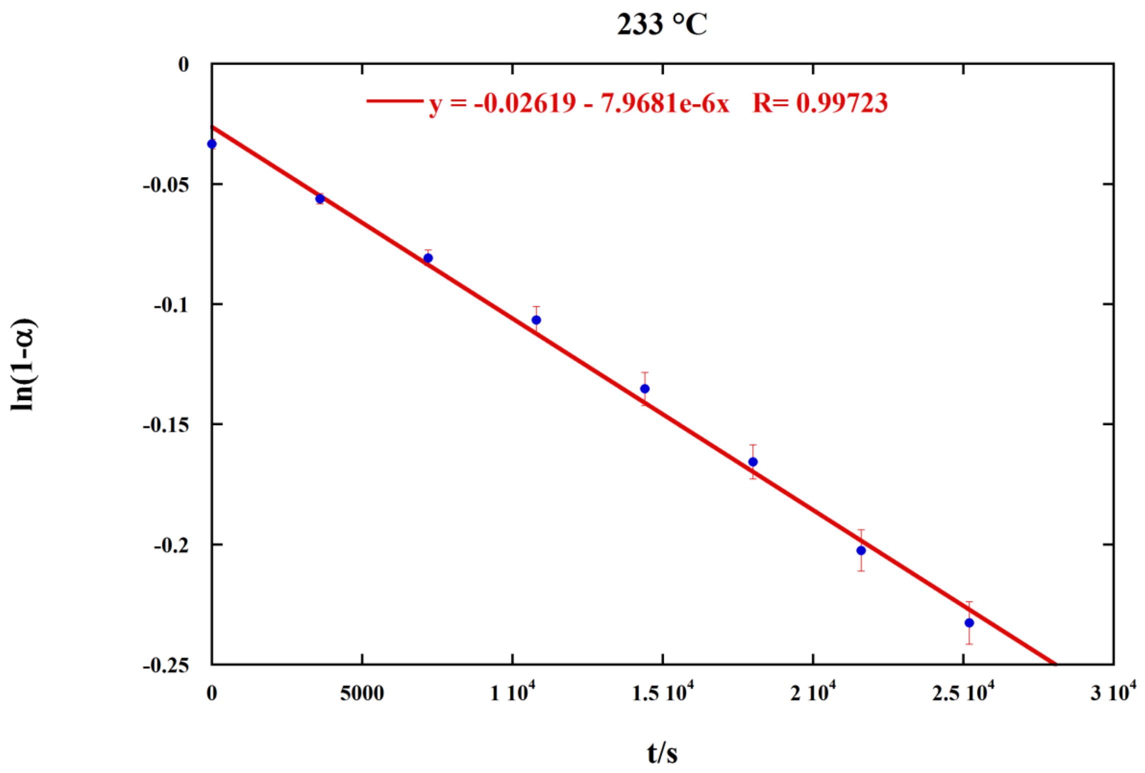
233 °C
y = -0.02619 - 7.9681e-6x R= 0.99723
ln(1-α)
0
-0.05
-0.1
-0.15
-0.2
-0.25
0
5000
1 10^4
1.5 10^4
2 10^4
2.5 10^4
3 10^4
t/s

**Fig. S7**

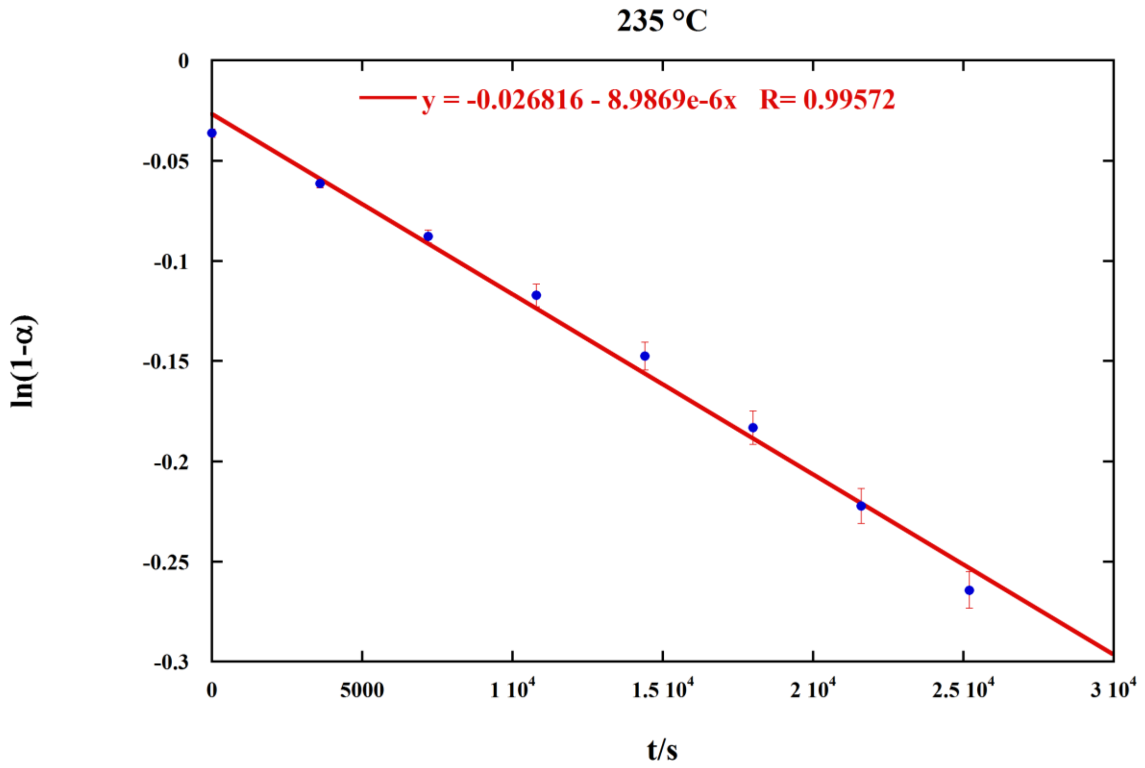
235 °C
y = -0.026816 - 8.9869e-6x R= 0.99572
ln(1-α)
0
-0.05
-0.1
-0.15
-0.2
-0.25
-0.3
0
5000
1 10^4
1.5 10^4
2 10^4
2.5 10^4
3 10^4
t/s

**Fig. S8**

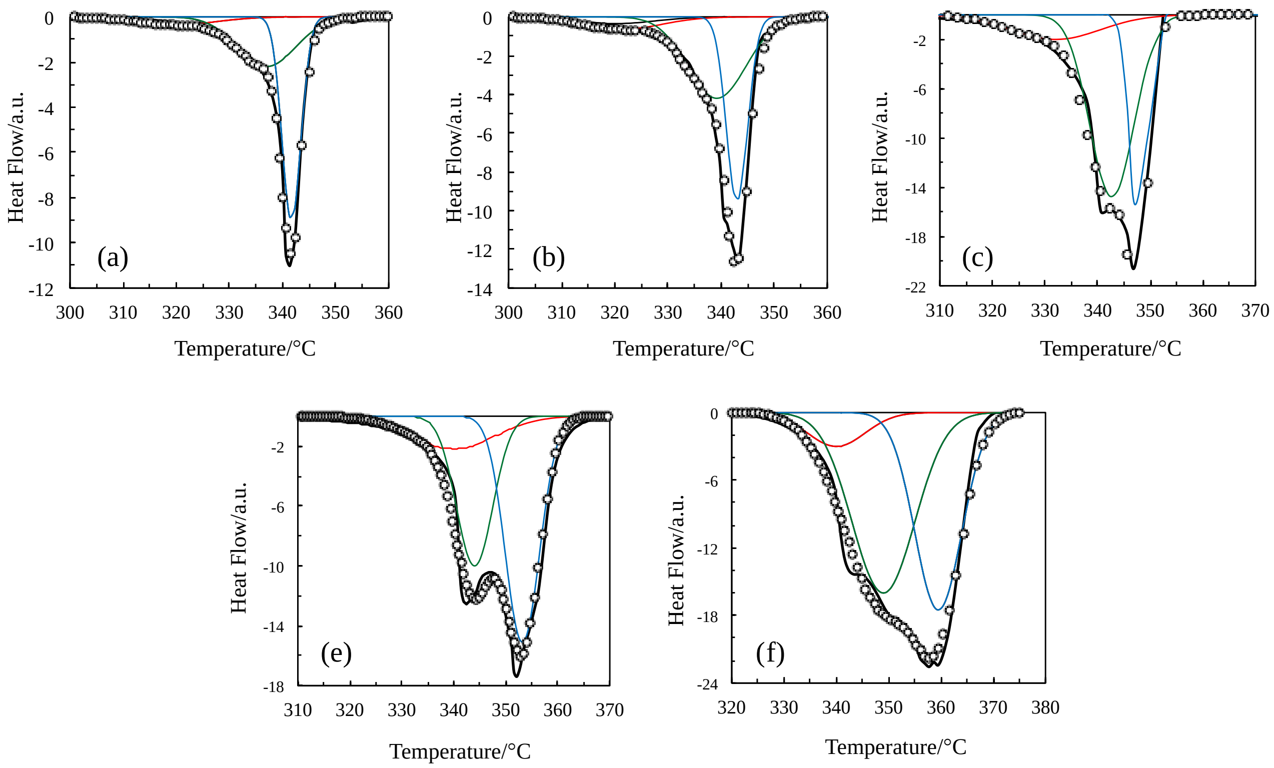


**Fig. S9.** Deconvolution of the DSC peak recorded at each constant heating rate for FAPI decomposition.
